# Pioneering, prodigious and perspicacious: Grunya Efimovna Sukhareva’s life and contribution to conceptualising autism and schizophrenia

**DOI:** 10.1007/s00787-021-01875-7

**Published:** 2021-09-25

**Authors:** David Ariel Sher, Jenny L. Gibson

**Affiliations:** 1 Department of Psychiatry, Warneford Hospital, University of Oxford, Oxford, OX3 7JX UK; 2 Faculty of Education, University of Cambridge, 184 Hills Road, Cambridge, CB2 8PQ UK

**Keywords:** Sukhareva, Autism, Schizophrenia, Russia, Psychiatry, Asperger

## Abstract

Grunya Efimovna Sukhareva’s seminal role in being the first to publish a clinical description of autistic traits in 1925, before both Kanner and Asperger, has been revealed relatively recently. Nevertheless, Sukhareva’s work is little known and largely unrecognised beyond Russia. Amidst calls for greater recognition of her pivotal contribution in the genesis of autism conceptualisation and categorisation, this article provides a biographical and historical background. Sukhareva’s wide-ranging psychiatric work is adumbrated and her pioneering efforts in conceptualising both schizophrenia and autism are elucidated. The article reflects on possible explanations for the belated and incomplete recognition of Sukhareva’s role. The current article indicates how Sukhareva’s work was ahead of its time in reflecting modern criteria for autism diagnoses and in its focus on female case studies. Sukhareva’s somewhat precarious position as a foremost psychiatrist condemned in the Stalinist years for being anti-Marxist is explicated. The article outlines further directions for academic research on Sukhareva’s work and contributions.

## Introduction

In the 1960s, Dr Nancy Rollins, an American specialist in child and adolescent psychiatry, perfected her knowledge of the Russian language and Soviet psychiatric literature. Thus armed, she embarked on a 4-month investigative excursion to the USSR to examine Soviet theory, treatment and diagnoses of psychiatric disorders among children and adolescents. She would later present this to a Western audience [1, 2]. In Child Psychiatry in the Soviet Union, the literary result of her endeavour, Rollins described encountering Professor Grunya Efimovna Sukhareva in 1968. It seemed that Rollins was deeply moved by this encounter, and she was particularly effusive in her praise of Sukhareva. She wrote that Soviet child psychiatrists, particularly those near Moscow, “tend to follow the grouping of their beloved teacher, Grunya Efimovna Sukhareva.” “The privilege of knowing her” Rollins continued, “was perhaps the most valuable experience I had in the Soviet Union.” Rollins met Sukhareva when she “…was already in her seventies…The qualities I saw in her of warmth, compassion, and wisdom made an enduring impression. The first two were evident in the way in which she handled patients and in the devotion she inspired in her staff, trainees, and former trainees I met in other parts of the country.” Assessing Sukhareva’s psychiatric contributions, Rollins wrote “Her psychiatric interests have been extremely broad. She has made contributions to the organization of psychiatric services, research in schizophrenia and epilepsy, and the training and teaching of child psychiatrists and psychoneurologists” [3, p. 73]. Sukhareva is perhaps now best known as the first academic to delineate clinical portraits of autistic boys in a Russian journal in 1925 [4] and a German journal in 1926 [5]. This was some two decades before Kanner and Asperger published their seminal papers on autism [6–8]. Yet despite this achievement, Sukhareva’s work and papers have remained relatively obscure outside Russia. Scholars have intimated that Sukhareva was ignored and that her articles may have been usurped by others [5, 6, 9]. Others argue that her publications require further historical study [10–12].

## Methods

Sukhareva’s work has mainly reached English-speaking audiences through translations of two of her key articles [5, 12]. Whilst these are inordinately useful, they were not aimed at providing an account of her life and contributions to psychiatry. An additional paper on Soviet psychiatry and the origins of ‘sluggish schizophrenia’ considered Sukhareva’s writings, but this was not its primary focus [13]. Similarly, whilst an informative, albeit brief, outline of Sukhareva’s descriptions of autistic male children and a comparison to the DSM-5 has been presented in the Nordic Journal of Psychiatry [6], this did not aim to provide detailed biographical contextual information or consider Sukhareva’s account of autistic girls and her contributions to understanding schizophrenia. Indeed, the authors called for wider recognition of her achievements. In this context, an English-language composition on Sukhareva’s life, background, and a broader focus on her psychiatric contributions, including both autism and schizophrenia categorisation and conceptualisation is pressingly needed. This paper aims to bridge this literature gap. This paper’s survey of Sukhareva’s work and contributions commenced with a literature search using PubMed, PsycINFO, Google Scholar, the iDiscover University of Cambridge search platform and the University of Oxford’s SOLO search engine (using the words; Sukhareva; Ssucharewa; autism; psychiatry). Whilst the search yielded Sukhareva’s own papers, we discovered very little material in mainstream English-language psychiatric literature on Sukhareva, aside from several exceptions which, as noted above, did not focus on Sukhareva’s overall contribution to psychiatry [5, 6, 12, 13]. We, therefore, perused Russian language literature concerning Sukhareva. To enhance rigour, the authors cross-checked material gathered from Russian-language publications with a psychology lecturer based in the United Kingdom, who speaks Russian as a first language. The lecturer, who studied clinical psychology at the State University of Saint-Petersburg and now resides in the United Kingdom, specialises in psycholinguistics and social cognition in autism. Her input is noted in the acknowledgements section. To widen the scope of our review of literature on Sukhareva, we also searched for mentions of Sukhareva by other prominent psychiatrists. Scans of Kanner’s papers referring to Sukhareva and Sukhareva’s articles in several journals which have since ceased publication were accessed through librarians at the University of Oxford’s Bodleian Library. A copy of Asperger’s original 1942 Habilitationsschrift was purchased from the Vienna University archives to ascertain whether the thesis cited Sukhareva. As such, the overall method employed can be conceptualised as consisting of three stages: examining Sukhareva’s own publications, exploring primary sources (i.e. literature published whilst Sukhareva was alive that directly pertained to her), and finally, exploring secondary literature on this topic (i.e. articles providing historical accounts of this topic). This approach facilitates synthesis of a range of material in forming a broad account of Sukhareva’s life and contributions. It also affords making suggestions for future research and for accounts which chart the genesis of autism conceptualisation and categorisation.

## Biography

Sukhareva was born to Chaim Faitelevich and Rakhila Iosifovna Sukharev in Kiev, then part of the Russian Empire, on 11 November 1891 [11]. In 1915, Sukhareva graduated from the Kiev Medical Institute. She worked in the institute’s epidemiological unit for 2 years. She then served as a psychiatrist at the Kiev Psychiatric Hospital from 1917 to 1921. During this period, in 1919, Sukhareva was also appointed head of the Defectology Department at the Institute of Mental Health of Children and Adolescents [14]. Sukhareva often held several prominent positions concurrently. In 1921, at age 30, Sukhareva established a school for children affected by psychiatric difficulties (she titled this a ‘hospital-school’) at Moscow’s Psychoneurological Department for Children, which was directed by Professor Mikhail Osipovich Gurevich. Children and adolescents would receive neuropsychiatric treatment here. It appears evident that Gurevich was Sukhareva’s primary mentor [11]. Gurevich is listed as the ‘supervisor’ on several of Sukhareva’s papers, including on her seminal 1926 paper [5] and it is clear that they worked closely. Sukhareva also composed sections on ‘schizoid psychopathies’ in a volume edited by Gurevich [4]. Gurevich had lived in Germany and worked under Kraepelin and Gannushkin [11]. As shall be seen further in this work, both Gurevich and Sukhareva worked in an increasingly repressive environment and were both forced to endure severe criticism from the Soviet authorities and like-minded academics. Sukhareva’s observations at Moscow’s Psychoneurological Department for Children later resulted in scholarly published articles. From 1928 to 1933, Sukhareva served as an Associate Professor at the First Moscow Medical Institute. She was head of the Psychiatry Department at the Kharkov Psychoneurological Institute from 1933 to 1935. Sukhareva also served as consultant and director at the Kashchenko Moscow Psychiatric Hospital from 1931 to 1951. Her work during this time laid the groundwork for the network of sanatoria and neuropsychiatric institutions across the USSR that Sukhareva would eventually create [14]. In 1935, Sukhareva successfully defended her doctoral dissertation and established the Child Psychiatry Department at the Central Institute for Postgraduate Medical Education, where she served as head, until 1965. During this time, several generations of child psychiatrists trained under Sukhareva and her colleagues. In 2017, Goryunov, Lazareva and Shevchenko wrote of Sukhareva, “It is difficult to overestimate her contribution to the training of personnel for scientific and medical institutions of the country” [14]*.* Apart from supervising several generations of clinical trainees, over 30 doctoral dissertations were completed under Sukhareva’s supervision. Sukhareva was able to instil leadership qualities in students and her disciples went on to found psychiatric schools of their own and occupy headship positions in Russia’s primary psychiatric hospitals and professional bodies.

From 1938 (by which time Sukhareva was already a professor) to 1969, Sukhareva headed the Psychosis Paediatric Clinic at the Russian Soviet Federative Socialist Republic’s (RSFSR) Institute of Psychiatry. During World War II, Sukhareva was evacuated to Tomsk, alongside the staff of Moscow’s Institute of Psychiatry. On the grounds of the Tomsk Psychiatric Hospital, a temporary ‘evacuation hospital’ was co-ordinated. Together with prominent academics and clinicians, Sukhareva treated those suffering from craniocerebral trauma and assisted with diagnosing patients. Several studies on post-traumatic disorders and other psychiatric implications of warfare were published. A paper by Sukhareva based on these experiences was published in English in 1947, and was titled ‘Psychologic disturbances in children during war’ [15]. In it, Sukhareva referred to the paediatric department of Moscow’s Kashchenko Hospital, whose sanatoria for male and female children were directed by her colleague E.A. Osipova, with whom Sukhareva would later publish several papers [15–17]. Sukhareva’s paper on psychologic disturbances indicated an increase in psychiatric conditions due to exposure to infection, toxins and trauma caused by the occupying German forces. After her return to Moscow and even following the conclusion of World War II, Sukhareva served as a consultant to the military hospital departments of Moscow’s Kashchenko Psychiatric Hospital, where she also supervised psychiatric research. Even in the increasingly restrictive and often punitive working environment she endured as a psychiatrist in the USSR (as detailed in the sections below), Sukhareva managed to remain in contact with other key pioneers of autism categorisation. Notably, Sukhareva corresponded with Leo Kanner, and as outlined in the coming sections, she succeeded in sending him copies of her seminal scholarly publications [18]. Sukhareva was a board member of the Soviet Union, Russian Federation and Moscow Society of Neurologists and Psychiatrists and chaired the childhood division of the latter organisation. Sukhareva was awarded the Order of Lenin, the Order of the Badge of Honour, and the title Honourable Scientist of the RSFSR. She never had any children. On 26 April 1981, Sukhareva passed away in Moscow after battling a long illness. At age 90, she was laid to rest at the Vostryakovskoye Jewish cemetery alongside her parents. Her sister, Maria, a paediatric doctor specialising in infectious diseases, is buried in the same cemetery [11].

## The first clinical account of autistic children: Sukhareva’s 1925 paper

Almost 20 years before Kanner and Asperger, Sukhareva published a comprehensive clinical description of six boys aged between 2 and 14 who had spent approximately 2 years at her ‘hospital-school’ and displayed symptoms of what is now titled autism spectrum disorder (ASD). Sukhareva’s article was first published in 1925 in the Russian-language journal Questions of pedology^1^ and child psychoneurology [4] and in 1926 Sukhareva published a German translation titled Die schizoiden Psychopathien im Kindesalter, in Monatsschrift für Psychiatrie und Neurologie, a German-language psychiatric and neurological journal [5]. The journal spelled Sukhareva’s name as Ssucharewa, potentially as a Germanic transliteration of the Cyrillic.

In her discussion of ‘schizoid psychopathy’, Sukhareva referred to Ernst Kretschmer and Eugen Bleuler’s conceptions of the phenomenon. In 1959, in a Russian composition which awaits publication into English, Sukhareva opted for the term ‘autistic (pathological avoidant) psychopathy’ rather than ‘schizoid psychopathy’ [6, 19]. In later years, psychiatrists used the term schizoid to refer to autistic children in a similar fashion to Sukhareva’s use of the terms. For example, in a substantial volume published in 1995, noted psychiatrist Sula Wolff explained that she used the term ‘schizoid’ broadly, and that such ‘affected children’ were akin to those discovered by ‘Ssucharewa (1926) and Asperger (1944)’ although the term also overlapped with ‘more seriously handicapped [sic]’ categories [20]. Sukhareva’s use of the term ‘autistic (pathological avoidant) psychopathy’ has echoes in today's debates about the relation of pathological demand avoidance (PDA) to the autism spectrum [21, 22], although it does not appear that PDA and ‘autistic (pathological avoidant) psychopathy’ are necessarily synonymous.

Sukhareva’s 1926 article is distinguished by its sympathetic and empathetic tone. She tended to focus on the incremental successes that children made in development since their admission to the institution. Talents and intellectual gifts are stressed in almost all her cases. She noted of a child “He is deeply emotional and very attached to his sister. If a letter from her arrives, he will hide in a corner to read it on his own rather than in the presence of witnesses, waiting patiently until he is left alone. Truthful and pedantic, he always takes a principled viewpoint…He has deep feelings for the beauties of nature…He is musically gifted, has a good ear, a rich musical memory…” Another example: “He is also an able artist, and the drawing teacher, himself an artist, assessed him as artistically highly gifted.” She noted about one child “he writes comprehensive articles for the children’s newspaper, some of which demonstrate excellent literary gifts (a journalistic style with a touch of humour).” Sukhareva had a positive view of the ability of children to adapt successfully if afforded favourable conditions: “The children's psychiatrist, observing sick children in life, in the family and helping them to adapt at school, was able to prove how important the social environment, the correct upbringing and education of a child [is] to stimulate his compensatory opportunities”; this reflected Sukhareva’s evolutionary-biological concept of mental illness. Sukhareva’s 1926 paper balanced these descriptions with comments relaying unconventional behaviour and challenges faced by children (e.g. “Writes notes with absurd contents to his doctors and child carers, put a card into the bag of one of the doctors which reads: “Honorary member of the society of fried dogs”; in another note he announces that he is giving a “lecture on all the nutrients contained in cotton wool!”) [5].

Notably, in her individual descriptions, Sukhareva summarised two children as ‘autistic’ or featuring ‘autistic reactions.’ In her overall summary, in a section titled ‘An autistic attitude’, she wrote: “All affected children keep themselves apart from their peers, find it hard to adapt to and are never fully themselves among other children…All these children manifest a tendency towards solitude and avoidance of other people from early childhood onwards; they keep themselves apart, avoid communal games…” Sukhareva described children with attributes that modern clinicians would immediately recognise as ‘autistic’. For example, she wrote that five out of the six children she described in 1925 displayed ‘a tendency towards automatism’ and that this manifested “as sticking to tasks which had been started and as psychic inflexibility with difficulty in adaptation to novelty.” She also referred to “a certain flatness and superficiality of emotions” and a “lack of facial expressiveness and of expressive movements” in her overall summary of cases. Referring to unusual talents in her notes on her cases, she also observed a proclivity for repetitious and unusual behaviour. She observed “strong interests…pursued…exclusively” in one of her clinical cases, “frequent repetitions of the same word” and ‘unusual’ sensitivity to noise [5]. Sukhareva provided details on physiological and anatomical features and her accounts are so meticulously detailed with anecdotal description of notable behaviour/occurrences, that readers are endowed with the sense of being able to recognise the children concerned, should they chance upon them.

## Acclaim for the style and ‘modernity’ of Sukhareva’s 1926 paper and her other work

Sukhareva’s 1926 descriptions of autistic children have earned considerable acclaim. Virtually all authors appraising her work offered particularly laudatory comments on its style and it being in accord with modern conceptions of autism. Edinburgh-based psychiatrist Sula Wolff, [5] who published an English translation of Sukhareva’s 1926, declared that Sukhareva summarised the children’s characteristics ‘admirably’ and that her description was ‘marvellous’ [23]. Russian psychiatrists at the Mental Health Research Centre, and the Russian Medical Academy of Postgraduate Education, both in Moscow, wrote that Sukhareva’s works “were distinguished by simplicity, clarity, consistency and systematic presentation of the material” [14]. Manouilenko and Bejerot [6] affirmed Sukhareva’s descriptions were ‘structured, elegant, detailed’ and ‘vivid’ and Sukhareva’s conception of ways of helping autistic children were strikingly ‘modern’. This paralleled Wolff’s comment that Sukhareva’s discussions before and after the case reports “contain ideas which are strikingly up-to-date”. Interestingly, Wolff's translation of Sukhareva’s paper describes the cases she portrays as having, in summary, a diagnosis of “Personality disorder: schizoid (eccentric)” [23]. It is worthwhile noting that Sukhareva’s diagnosis has relevance to contemporary research concerning the relationship between autism and borderline personality disorder (BPD), a condition which is more frequently diagnosed in females [24]. A study titled ‘The overlap between autistic spectrum conditions and borderline personality disorder’ found that similarly to autistic participants, BPD participants featured elevated autistic traits and a powerful urge to systemise, which indicated an overlap between autism and BPD [25]; this is also reflected in more recent studies [26]. Similarly, it has been revealed that BPD patients featured higher autistic traits than control group participants and that autistic traits had a significant impact on some clinical features associated with BPD, such as suicidality [27]. Researchers have argued that difficulties with empathy and theory of mind are overlapping features in BPD and autism [28]. A modern, impassioned debate on whether BPD should be diagnosed or not [29] also has resonance in the context of Sukhareva’s summary diagnosis.

Writing in the Journal of Pediatric Neurosciences, Posar and Visconti [30] declared: “The description of these cases is of an amazing precision and modernity: just think of the fact, for example, that Grunya Efimovna Sukhareva emphasized the importance of the presence of sensory abnormalities, which only recently regained their proper weight in the description of ASD in the DSM-5.”

The striking parallels between Sukhareva’s clinical descriptions and the DSM-5 are outlined point-by-point by Manouilenko and Bejerot [6]. For example, the DSM-5 specifies social deficits across different contexts should be present for an ASD diagnosis and Sukhareva referred to ‘flattened affective life’ and “Lack of facial expressiveness and expressive movements.” Corresponding to the “stereotyped or repetitive behaviours, restricted interests and sensory sensitivities” in the DSM-5, Sukhareva referred to “Repetitive questioning; talking in stereotypic ways; strong interests pursued exclusively.” Paralleling the DSM-5’s “Hyper- or hypo-reactivity to sensory input”, Sukhareva noted sensitivity to noise and smell. Sukhareva also referred to the child’s parents and other relatives, a nod to a belief, current even then [5], in the heritability of autism [31]. Basing herself on histopathological research, Sukhareva indicated her belief in an anatomical substrate of autism; an ‘inborn abnormality’ of the cerebellum, basal ganglia and frontal lobes [14] Sukhareva’s assertions are now backed by modern neuroimaging research which has linked these brain areas to autism [6, 32–34].

Sukhareva’s ‘hospital-school’ featured classes in woodwork, art and gymnastics and children were afforded opportunities to play musical instruments. The school also strove to impart motor and social skills to the students. Teachers worked in close concert with doctors and adopted a highly individualised approach, which considered each child’s condition and diagnosis. Sporting events, matinees and children's concerts were held. Girls were offered occupational therapy in the form of sewing and artistic embroidery, whilst boys were offered carpentry and artwork. Children were empowered with responsibilities, for example some were charged with feeding birds and animals in the hospital-school’s ‘zoo’ corner, whilst others, under specialist guidance, performed agricultural work and tended to flowers. Berries, fruits and vegetables were harvested exclusively by the children, who also undertook record-keeping and distribution to the departments [35]. The heterogenous array of activities were subsidised by the government and in this context, researchers could observe children in a multiplicity of environments and classes. This undoubtedly assisted Sukhareva in her observations which were meticulously detailed and included comments on children’s familial and scholastic background, relationship with family, idiosyncrasies and talents. The hospital-school served as a conduit for social integration into mainstream schools.

Like her papers on autism, when Sukhareva’s other works were translated into English, they received wide praise. For example, Australian academics praised Sukhareva’s paper ‘The problem of the classification of mental retardation’, published in 1972 [36]. “We have been impressed with Suhareva’s classification (Suhareva, 1972)” Pitt, Roboz and Plant [37] declared. Several years later, other Australian researchers opined “…it therefore seems likely that future refinements in classification, leading eventually to international usage, will be along the lines of Suhareva’s (1972) classification with modifications of the type suggested by Pitt et al. (1974)” [38].

## Focus on females

Another way in which Sukhareva’s work was ahead of its time was in its focus on females. Wolff made no mention of Sukhareva’s sequel to the 1926 paper that she translated. In 2020, a paper titled ‘The first account of the syndrome Asperger described? Part 2: the girls’ was published in the Journal of European Child and Adolescent Psychiatry [12]. Translated by Charlotte Simmonds, the paper was the second part of Sukhareva’s 1926 article ‘Die schizoiden Psychopathien im Kindesalter.’ Part two was titled ‘Die Besonderheiten der schizoiden Psychopathien bei den Mädchen’ (‘The particular features of schizoid psychopathies in girls’) and was published in 1927, in Monatsschrift für Psychiatrie und Neurologie, the same German-language journal as Sukhareva’s seminal 1926 paper. The sequel comprised five case studies of girls with autistic symptoms and discussed sex differences in what is now known as ASD. Simmonds noted that for much of the twentieth century, prominent psychiatrists, such as Asperger and van Krevelen, maintained that autism occurred virtually exclusively in males; Asperger attributed autistic symptoms in girls to postencephalitic conditions. Sukhareva’s arguments in 1927 indicated otherwise and she asserted that often “people who have recovered from encephalitis and have motor skill disorders, for example, display no autistic disposition.” Critically, Simmonds noted how over 90 years ago, Sukhareva delineated the sex differences in autistic symptoms that are only being adumbrated today: “The female differences being described now: greater affect dysregulation, less idiosyncratic interests; and the similarities: autistic disposition, low or absent affective empathy, unimpaired cognitive empathy, systemising thought processes, motor skill deficits, were laid out by Sukhareva in 1927.” In her opening paragraph, Sukhareva noted that her report should be viewed as a continuation of her ground-breaking paper ‘Schizoid psychopathies in childhood’, published in 1925 in Russian [4] and a year later, in German [5]. Both papers concern children whom Sukhareva would have had an extended period of time to observe, for 2–4 years. Similar symptoms manifested themselves in the females as in the males. Children featured repetitious, stereotypical behaviour (one answered all questions with the refrain “Don’t ask me, I won’t tell you anyhow, it’s my secret.”) flattened emotion, (“all affective motions remain externally cold and weakly expressed”) and inflexibility (“an idiosyncratic, inflexible, and hyperbolic sense of justice”; “tendency towards autistic responses: introversion, reticence, little sociability”; “emotional coldness and inertia of the affective responses”) [12].

In concord with modern conceptions of systemisation amongst autistic children [39], Sukhareva referred to “a definite tendency towards systemisation”, relating that her second female case study answered the question ‘What is a fork?’ with the answer “An object which is made of something such as iron and has several appendages.” Children did not express significant emotion and had difficulty in socialising. ‘Case 3’ was described as “An emotionally flat girl: has no longing for her relatives, no close girl friends…When among children, she keeps herself apart, she doesn’t participate in group play.” ‘Case 4’ related “I have never had strong feelings”, “Nothing particularly moves me”, “I have no close girl friends, I don’t like being close to people” whilst ‘Case 2’ affirmed “I find all girls unpleasant, I don’t love anyone.” Sukhareva noted about her final female case study “Emotional contact cannot be made with her immediately and can only be made with difficulty.” In all these cases, Sukhareva wrote that a diagnosis of schizophrenia could be ‘ruled out’ as schizoid symptomatology had been manifest since birth and had not deteriorated; the significant improvements witnessed in the course of the children’s lives were also not typical of schizophrenia. Sukhareva also viewed an exogenous explanation as most unlikely, for such explanations would not explain the full picture of schizoid psychopathy.

As reflected in modern studies finding that autistic children often do not engage in or comprehend pretend play [40–42] and often systemise [39], Sukhareva made the striking observation of her fifth case study “She did not like dolls and broke them to find out what was inside; preferred active games with boys.” Like her account of the male children, Sukhareva’s writing sensitively emphasised the female children’s talents, noting that one child was “musically gifted (ability to compose)” and in her account of her fifth case study “She gives a very fine rendition of emotional experiences on stage; she has a delicate sensitivity for the beauty of nature and books. She has intense intellectual interests which she satisfies by means of reading.”

Notably, Sukhareva did not shun accounts which portrayed her institution’s struggles to effectively assist some of her charges. By honestly relaying unflattering details, an enhanced sense of trustworthiness surrounds her descriptions. For example, Sukhareva wrote of one girl: “She finds everything here very unpleasant, everything here meets with censure…To the question: “What do you dislike?” she gave the answer: “I dislike everything and everyone here is bad.”” Overall, Sukhareva wrote that her studies of both female and male children with schizoid psychopathy revealed two forms of symptomatology; (a) fundamental symptoms of a schizoid “psychopath’s” psyche and (b) accessory symptoms that often appear but not consistently. The basic symptoms were delineated as “1. the autistic attitude, 2. the ambivalence of the thymopsyche, 3. the idiosyncratic thought processes: tendency towards the abstract and formal; automatism, and 4. symptoms of motor skill deficiency: angularity, clumsy movements.” Accessory symptoms included paranoia/persecutory delusions; psychasthenic syndrome (including feeling inferior and insecurity); ‘catatonoid’ symptoms including ‘increased suggestibility’ and/or ‘pronounced negativism’; ‘psychomotor disorders’ including tomfoolery, capriciousness, automatism, and proclivity for stereotyped movements [12].

Importantly, Sukhareva observed that “The symptom of autistic disposition is equally characteristic of both sexes.” She wrote about differing levels of autism “In three of the cases described, we are talking about a strongly pronounced autism, in two others, it is a low or elective sociability. All these girls appear introverted, reticent, not particularly approachable. All were “loners” from early childhood and mention it themselves” [12]. Sukhareva wrote that the clinical picture ‘overlapped’ between the sexes in their primary traits. However, she noted that girls with schizoid psychopathology manifested greater affective disturbance than boys; Sukhareva ascribed this to what she termed the more potent and “volatile affectivity of the female psyche.” Girls also manifested greater levels of negativism, with ‘hysteroid’ attributes. Sukhareva indicated that hysterical symptomatology was more common in girls than boys but cautioned against a ubiquitous tendency amongst clinicians to confuse schizoid psychopathy with hysteria; Sukhareva provided a detailed explication for why these should not be conflated or confused. She argued that people with hysteria have an affinity for social settings where they can ‘exhibit’ themselves, whilst the ‘schizoid’ children she described tended to be loners and were not very sociable. She stated that the ‘schizoid’ girls were more independent, were ‘firmer’ in their intentions and were not easily ‘influenced’, as they did not have the required ‘emotional receptivity’. The ‘schizoid’ girls did not have the ‘reactive lability and suggestibility’ of people with hysteria. Additionally, the potent affectivity which Sukhareva deemed characteristic of people with hysteria was not present in the ‘schizoid’ girls, who exhibited a certain ‘coldness’. Finally there were no ‘somatic stigmas’ characteristic of hysteria present in the ‘schizoid’ girls. For example, there were no ‘attacks’ or ‘somatosensory disorders’ [12]. Conversely, Sukhareva found that schizoid thought patterns were less pronounced in girls, who demonstrated less of a proclivity for abstract and schematic thinking. Similarly, motor skill deficits including facial expressions and expressive movement were less manifest amongst the females [12]. In 1930, Sukhareva elucidated the life projection for those with schizoid psychopathy in a Russian paper that awaits translation [43]. In this paper, a triad of key biological insufficiencies were delineated; psychomotor impairment (e.g. unusual angular movements, automatism); emotional impairment (e.g. poor affective attachment to others); and divergence in associative work and thinking (e.g. predilection for abstract thought and inflexibility or rigidity in thinking). It is worthwhile noting that in modern conceptions of autism, it is acknowledged that autistic children can form secure attachments. Indeed 47% of autistic children would be classified as secure when using the Strange Situation Paradigm [44]. Furthermore, it is now argued that alexithymia, which is associated with difficulty in recognising affective information in others and an inability to express and determine one’s own emotions, is a distinct condition from autism [45]. Whilst approximately half of autistic people meet the criteria to be deemed alexithymic, an almost equal number do not. Indeed, whilst having socio-emotional difficulties has long been considered a key autistic trait, it is now opined that such difficulties are actually caused by co-occurring alexithymia and not autism [46].

In the context of Sukhareva’s focus on autistic females, Simmonds has opined that ‘the Anglo-European world’ was left almost 100 years behind Russia in recognising the ‘female autism phenotype’ [11]. In this vein, it is troubling to note that in the opinion of scholars on this subject, misogyny likely contributed to preventing greater fame for Sukhareva’s pioneering papers [6, 11].

## Why did Asperger and Kanner not credit Sukhareva?

In his 1944 paper, Asperger wrote “The aim of this paper was to report on a personality disorder already manifest in childhood which to my knowledge has not yet been described” [7]. Given Kanner’s publication on autistic children in 1943, Uta Frith speculated in a footnote to her translation of Asperger’s work [47] that although Kanner’s ‘classic’ paper on autism was published a year prior, it “would not have come to Asperger’s attention during the war years.” Frith did not address why no mention was made of Sukhareva’s paper, which was published in one of the very few German journals in the field at that time [6]. Asperger’s omission has led some organisations operating on behalf of the autistic community to suspect Asperger of disingenuity. For example, the Canadian autistic society, Autism Canada, states in its history of autism; “Sukhareva’s 1925 article is impressive, but was unfortunately overlooked until recently. It is quite possible that the more famous Hans Asperger read it, but he never credited her when he published in 1944 about the autistic traits that would later bear his name” [48]. It has been stated that Asperger’s original postdoctoral habilitation thesis manuscript (upon which the 1944 paper was based) has not been found [49]. To preclude the possibility that Asperger cited Sukhareva in his habilitation thesis submitted to Vienna University in 1942 but not in his later 1944 article, the first author of the present article succeeded in obtaining a copy of Asperger’s original 110 page type-script ‘Habilitationsschrift’ with Asperger’s handwritten corrections, from the Vienna University archives. The archivist confirmed that it appeared obvious that the typescript was the basis for Asperger receiving the title ‘Dr. med. Habil’ on 10 March 1943. Although undated, records reveal that Asperger submitted his Habilitationsschrift on 19 October 1942, alongside his application for the ‘Dr. med. Habil’ title. We found that there are some ostensibly minor differences between the journal article and the original Habilitationsschrift, particularly in reference formatting. However, we found that no mention of Sukhareva was present in the text, although Asperger did cite articles from Monatsschrift für Psychiatrie und Neurologie, the same journal in which Sukhareva’s 1926 and 1927 papers were published [50]. Incidentally, both Sukhareva (in 1925) and Asperger (in 1944) cited Ernst Kretschmer’s well-known work Körperbau und Character [51].

It was only decades later, in 1996, that Sula Wolff published an English translation of Sukhareva’s 1926 paper in the journal European Child & Adolescent Psychiatry. The article was titled ‘The first account of the syndrome Asperger described?’ [5]. Wolff’s introduction and title alluded to her view that Asperger must have known about Sukhareva’s work (published in German almost two decades prior to his 1944 paper), and that Asperger appropriated Sukhareva’s work without referencing her. Unconventionally, Wolff concluded her introductory comments with a seemingly purposefully jarring question: “An unanswerable question remains: how was it that Hans Asperger, familiar as he was with Kretschmer’s work, did not apparently know of this paper?” she asked [5].

“On reading the paper which follows” Wolff declared, “it will at once be clear that the six boys described by Dr Ssucharewa some 70 years ago resemble very closely the children reported on by Asperger in 1944” [5]. Wolff’s paper was successful in drawing some attention to this issue. For example, in 2001, Eric Fombonne, the associate editor of the Journal of Autism and Developmental Disorders wrote “In 1944, Asperger described a syndrome which has subsequently been given his name, although there is evidence from the earlier European literature that clinical descriptions matching this disorder were available in the 1920s.” He then cited Wolff’s translation of Sukhareva’s 1926 paper [52]. Similarly, in 2005, Lorna Wing noted Wolff’s translation and wrote that Sukhareva preceded Asperger, by publishing her paper in 1926. Wing then stated “By the chances of history, Asperger’s name rather than Ssucharewa’s has become associated with the ‘‘syndrome’’.” [53]. In 2015, Manouilenko and Bejerot noted Wolff’s intimation that Asperger was aware of Sukhareva’s article when he wrote his 1944 paper. They indicated that without concrete evidence, this was speculatory [6]. Nevertheless, Manouilenko and Bejerot similarly implied that Asperger knew of Sukhareva but chose not to credit her. They wrote of the notability that Asperger cited a 1938 paper published in Monatsschrift für Psychiatrie und Neurologie, the same journal as Sukhareva’s 1926 paper and that the journal was one of only a handful of publications in the field at that time, meaning Asperger would likely have read it [6]. In his history of autism, Adam Feinstein related that psychiatrist Lorna Wing informed him: “No one is totally original….Asperger may have read Eva Sushareva’s [sic] 1926 paper”. Feinstein also noted that it was clear that Sukhareva delineated the key hallmarks of the syndrome many years before Asperger [54]. In a 2004 article on the history of autism, Wolff once again drew attention to this issue, noting “Asperger’s work was less systematic than Kanner’s.” Pointedly, she then used italicisation to emphasise Asperger’s omission: “*His* literature review too was incomplete” Wolff wrote. “He failed to mention the marvellous German account of 6 cases exactly like his own, described by Ssucharewa as “schizoid personality of childhood” in 1926” [23].

Elsewhere, Manouilenko suggested that Asperger read Sukhareva’s paper but decided not to cite it as he may have been precluded from citing a Jewish author. However, this seems somewhat unlikely, for Sukhareva was cited in German journals and volumes well into the Nazi period and during World War II [55–59]. Manouilenko also offered a more disturbing option, in that Asperger may not have wished to credit a Jewish academic in light of Asperger’s co-operation with Nazi scientists and involvement with the Nazi euthanasia programme [9]. This refers to Asperger’s signing of papers for at least two disabled children to be transferred to Vienna’s Am Spiegelgrund killing facility ‘hospital’ where they were subsequently killed, alongside his clinical appraisal for the Nazi regime of 35 children from the Gugging psychiatric hospital as being ‘uneducable’ and ‘unemployable’. Such labels were inexorably linked to unwilling ‘euthanasia’ [49, 60, 61]. Manouilenko’s rationale for Asperger’s omission is plausible, for by 1938 Asperger was signing his diagnoses with ‘Heil Hitler!’ and by 1940, Asperger was a member of a number of virulently anti-Semitic bodies, including the National Socialist German Physician’s League, the figurehead of the Nazi Party within the medical profession. Similarly, Asperger superfluously drew attention to his patients’ Jewish lineage, and in 1940 wrote of a boy named Ivo; “The only problem is that the boy is a Mischling of the first degree” Asperger’s unnecessary use of this label—which referred to individuals with one Jewish parent—was an exceedingly perilous and potentially fatal piece of information. Asperger also made some plainly anti-Semitic statements. Writing ‘Mischling’ on the first page of 9-year-old Marie Klein’s diagnostic report, he wrote that the way she spoke contrasted “to her quite Jewish character”. From Asperger’s ward in Vienna, Marie was sent to a children’s home and in February 1940 she was transported to the Wlodawa ghetto, from where children were taken in ‘Aktions’ to be murdered at Sobibor. In 1939, a 12-year-old Jewish girl, Lizzy Hofbauer, was admitted to Asperger’s clinic. Displaying trepidation and speaking of anti-Jewish persecution; (a reality in Nazi-ruled Vienna), Asperger claimed Hofbauer was schizophrenic and noted “For her age and race, conspicuously retarded sexual development”; evidence that he had internalised sexualised Nazi anti-Jewish stereotypes [49, 60, 61]. In this context, Manouilenko’s suggestion does not appear unjustified.^2^

Conversely, Kanner did cite and later write admiringly about Sukhareva [18]. However, he did not cite Sukhareva in his 1943 paper but in a 1949 paper (Problems of nosology and psychodynamics of early infantile autism [62]) and even then he did not refer to her 1926 paper but to a paper she had published in 1932 [63] (Kanner also later cited Sukhareva’s work on schizophrenia [64]). It has been opined that this does not mean that Kanner’s 1943 paper was inspired by Sukhareva’s work [30]. In recent years, it has been convincingly argued that both Kanner’s 1943 paper and Asperger’s 1944 paper were based upon the work, expertise, and ideas of Georg Frankl, a more experienced Jewish clinician than Asperger at the Vienna Kinderklinik and those of his wife, Anni Weiss [60, 65–67], although Asperger decided not to credit them. These assertions have recently been backed by concrete historical and documentary proof [68, 69]. Nevertheless, Sukhareva’s work was published a decade prior to Anni Weiss’ account of children with autistic traits, ‘Qualitative intelligence testing as a means of diagnosis in the examination of psychopathic children’ which only appeared in The American Journal of Orthopsychiatry in 1935 [70]. Similarly, recent evidence showing that Kanner expressed much admiration for Frankl’s work and intended for Frankl’s paper to be published before his own, in 1943 [68, 69] does not affect Sukhareva’s position in publishing the first clinical description of autistic children almost 2 decades prior, in Russian in 1925 [4] and in German in 1926 [5].

## Sukhareva’s other literary contributions

During the 1930s, Sukhareva developed what was subsequently referred to as an evolutionary biological conception of mental disorders whose significance to modern psychiatry was fully elucidated in Russian by Shevchenko in 2016 [71]. In her first two editions of ‘Clinical Lectures on Child Psychiatry’ (Volume 1) published in 1939 and 1955, [72, 73] Sukhareva provided an explication of essential elements of mental illness in adolescence and childhood. Critically, Sukhareva expounded that alongside the injurious elements typified in mental illness and preventative ‘defence’ mechanisms, progressive elements could also be manifest, which indicated the overarching continued evolution of cerebral and overall development. She also stressed the role of age-related issues and environmental influence and indicated that alterations to an individual’s social environment could improve ‘acquired’ psychopathy in a far more efficacious fashion than constitutional psychopathy [19]. Sukhareva provided an account of what is now titled attention deficit hyperactivity disorder (ADHD) in modern classifications [74]. In her discussion of schizophrenic pathology, Sukhareva indicated when defining ‘defect’ (in the context of deficit syndromes), that the concept overlaps with similar terms such as residual states and negative symptoms. This similarity makes defining ‘defect’ a difficult exercise. Defect symptoms included resistance to treatment, a correlation with cerebral structural changes, and an absence of progression of the deficits, which generally remain unchanged. Sukhareva described subtypes of the deficit syndrome as apathetic, apatho-abulic, asthenic and atonic [75, 76].

Using the length of exposure to a pathogenic agent and its aetiology as a foundation, Sukhareva categorised oligophrenia (a Soviet term referring to intellectual disability, sometimes referred to in outdated English literature as ‘mental retardation’) into three groups: (a) pathology caused by damage to parents’ reproductive cells; (b) those caused by harmful factors that occurred during the period of intrauterine development; and (c) those caused through damage to the central nervous system in the perinatal period or in the child’s first 3 years [36]. In her work on epilepsy, Sukhareva provided diagnostic criteria and accounts delineating paroxysmal night terrors, somnambulism, visual hallucinations and other related phenomena [72]. She also undertook considerable work on mental trauma and pathogenesis of somatogenic and psychogenic psychoses affected by war. Such work included a focus on children with viral diseases, rheumatism and congenital syphilis. Goryunov, Lazareva and Shevchenko [74] noted that Sukhareva contributed extensively to every branch of child psychiatry and deem her a founder of Russian child psychiatry. She was prodigious and authored over 150 scientific papers, six monographs, and a three-volume collection; ‘Clinical Lectures on Child Psychiatry’, which summarised her research in the field. These were published in 1955 [72] (first edition 1939 [73]), 1959 [19], and 1965 [77] This was a considerable achievement, considering their publication coincided with a period of Soviet subjugation of psychoanalysis, genetics and cybernetics.

## Political context of Sukhareva’s work

In the 1920s and 1930s, Soviet psychiatrists explored pathways for diagnosis of early stages of schizophrenia. Their task was hindered, for schizophrenia had a wide array of potential symptoms, including paranoid thoughts, hallucinations, verbal disorganisation and uncontrolled movement. However, patients might manifest none of these symptoms and still be diagnosed with schizophrenia. In this context, one noted Soviet psychiatrist lamented that whilst other ‘nosological units’ such as paranoia and neurasthenia were well defined and their definitions were still undergoing refinement, the nosological delineations of ‘schizophrenia alone’ seemed to grow incessantly [13, 78]. Similarly, schizophrenia’s broad definition made it difficult for psychiatrists to differentiate it from other conditions. Sukhareva’s supervisor in Moscow, M.O. Gurevich, had co-authored a 1928 textbook which defined schizophrenia as “an endogenous disease process characterised by progressive course of development of signs of splitting of the psyche and emotional dullness” [79, 80].

Schizophrenia caused great concern amongst Soviet health officials as by 1928 it accounted for one third of all patients in psychiatric hospitals. However, the boundaries of schizophrenia continued to widen. In the early 1930s, Dr Lev Rozenshtein declared he had discovered the existence of a distinct disease entity; mild schizophrenia [81, 82]. This pathologized relatively inconsiderable manifestations of anxiety, melancholy and lethargy. Rozenshtein was not alone. P.B. Gannushkin and V.A. Giliarovskii, both influenced by the well-known Russian clinician Sergei Korsakov, included patients without disorganised thinking, delusions, and hallucinations as still being under the umbrella diagnosis of schizophrenia. These patients were situated on the ‘less severe’ side of a schizophrenic ‘continuum’. Mood swings, fixation, or hysteria and a family history of schizophrenia often sufficed for a schizophrenia diagnosis. An article published by Oleg Kerbikov, a researcher working under Gannushkin, earned particular opprobrium amongst Russian psychiatrists for declaring in a chapter of a volume (edited by Sukhareva’s supervisor, M.O. Gurevich) that a patient had schizophrenia without any schizophrenic symptomatology [13, 80]. Conversely, the noted Soviet physiologist, Ivan Pavlov championed the notion that schizophrenia could be understood by being affected by the endocrinal system. The head of Leningrad’s Military Medical Academy, V.P. Osipov, espoused this view particularly forcefully [13].

It was in this confused environment that Sukhareva trained and practised psychiatry at the First Moscow Medical Institute, under Gannushkin, having completed her tenure as head of the child psychiatry department at Kiev’s psychiatric hospital [14]. Following her accounts of schizoid psychopathy in children, Sukhareva attempted to differentiate between schizoid psychopathy in children and childhood schizophrenia. Sukhareva observed a multiplicity of clinical manifestations and found that ‘simple schizophrenia’ was too crude a diagnostic label to account for the heterogeneity she observed. She found Kraepelin’s focus on process and outcome impractical for young children and that Bleuler’s ‘secondary characteristics’ were not evident. Thus, Sukhareva determined to monitor and measure the severity of onset of schizophrenic symptomatology and the ‘tempo’ of their development [13, 83, 84]. Observing 107 children, Sukhareva determined two discrete forms of schizophrenia amongst the children. One subset of children manifested an unhurried but unremitting prepubescent commencement of schizophrenia. These children had a family history of schizoid psychopathy or schizophrenia, displayed reticence and difficulty in socialisation with peers, featured clumsy movements and were particularly sensitive. Sukhareva labelled this form ‘sluggish’ (or ‘continuous’) schizophrenia and hypothesised that its causation was attributable to hereditary influences. In the second group Sukhareva studied, children featured a delayed onset of symptoms, which were only exhibited during or after puberty. Another dissimilarity to the first group was that the second group’s schizophrenia did not progress at a steady tempo; there was fluctuation between sudden eruption of symptoms and periods of remission. The condition developed suddenly and cataclysmically. Sukhareva labelled this latter form ‘rapid’ (or ‘paroxysmal’) schizophrenia and theorised that it was caused when a child with schizophrenic heredity was exposed to an external factor such as trauma [13, 85]. Although Zajicek referred to two varieties of schizophrenia delineated by Sukhareva, Goryunov et al. [14] stressed a third variant that Sukhareva referred to a mixed form where a ‘sluggish continuous flow’ was evident alongside occasional paroxysmal flare-ups.

Meanwhile, several professors (including A.S. Shmar’ian, whom Sukhareva had worked with in Tomsk) began to severely criticize the status quo of Russian psychiatry. Osipov wrote especially censorious animadversions against Rozenshtein’s ‘mild schizophrenia’ and opined that Rozenshtein had irresponsibly advanced introversion into a condition which had spearheaded countless Soviets being thoughtlessly diagnosed as having ‘schizophrenia’. He postulated that the wide diagnostic latitude which ‘mild schizophrenia’ afforded had led to severe abuse by unethical officeholders, who ensured subordinates whom they had skirmished with were expediently diagnosed with mild schizophrenia [13].

Zajicek held Sukhareva’s work to be critically important in providing a ‘unified theoretical model’ to chart a path forward for Soviet psychiatry in this highly politicised and fraught juncture of Soviet psychiatry [13]. In 1935, Sukhareva published the article ‘Towards the problem of the unity of schizophrenia’ [13, 86]. Here, she posited that schizophrenia was not an arbitrary umbrella term for several disparate diseases. Rather, she indicated that schizophrenia was a unified, singular category and the heterogeneous array of its forms could be categorised and observed through her approach of monitoring the condition’s tempo, which would reveal the different forms of schizophrenia. Sukhareva’s work was of pivotal importance in synthesising disparate Soviet thought and research indicating the importance of psychological trauma, the patients’ heredity and the endocrine system. It was also acutely needed as it moved away from the divisiveness that Rozenshtein’s mild schizophrenia concept had engendered in Soviet psychiatry.

Nevertheless, Sukhareva faced unenviable challenges, for in preparation for the All-Union Society of Neuropathologists and Psychiatrists Congress in Moscow in 1936, Osipov had commenced efforts to utilise the congress to publicly renounce Rozenshtein’s mild schizophrenia research. Osipov was aided when in the summer of 1936, the Communist Party passed a resolution prohibiting using psychological methods in education and workplaces and thereby effectively terminating psychological research and practise in Russia. Osipov opined that the mild schizophrenia concept was guilty of pathologising minor disturbances as constituting schizophrenia [13]. As the congress approached, Sukhareva wrote two articles, designated ‘materials for the All-Union Congress’, in which she indicated that her approach of monitoring the pace of schizophrenic development, and determining whether a patient had sluggish or rapid schizophrenia could resolve the lack of consensus regarding whether mild forms of schizophrenia were disparate forms of their more severe counterparts and whether schizophrenia was endogenous or exogenous [13, 87, 88]. The congress was a highly acrimonious event and Osipov devoted time to criticising and repudiating Rozenshtein’s mild schizophrenia concept. Gannushkin and Rozenshtein were no longer alive; they escaped the fate suffered by their colleague, Brukhanskii, who was apprehended by police following the conference and dispatched to the Gulag, where he died in 1944 [13].

Sukhareva’s sluggish schizophrenia concept remained operative for decades as the standard term for milder, non-pronounced forms of schizophrenia, where it was also evident that ‘the unity’ of the ‘personality and a progredient process’ had suffered disruption [13, 89]. Sluggish schizophrenia was subsequently misused to imprison political nonconformists in the USSR following World War II [13]. Dr Andrei Snezhnevskii, a colleague of Sukhareva’s at the Institute for Advanced Medical Study, championed the use of Sukhareva’s sluggish, rapid and mixed schizophrenia conception and this remained the basis of schizophrenia’s clinical classification amongst Russian psychiatrists for many decades [13, 90].

## The Pavlovian Sessions

Another historical drama affecting Sukhareva related to the Pavlovian Sessions, held in Moscow in the 1950s. Stalin admired I.P. Pavlov’s higher nervous activity theory [91]. The view that humans might be moulded by the conditions surrounding them had obvious resonance for Communist ambition and Bukharin duly noted of Pavlov: “Ideologically, he works for us” [92]. Stalin quickly moved to institutionalise Pavlov’s teachings. As Windholz [93] argued, Stalin thereby hoped that a “new, tame and self-sacrificing Homo Sovieticus would emerge.” Ironically, Pavlov had been an outspoken critic of Communism.^3^

In 1950, the USSR Academy of Medical Sciences and the USSR Academy of Sciences held a joint session in Moscow, in accord with Stalin’s scheme. The session determined that yearly conferences should be convened to consider issues relating to Pavlov’s work. Thus, a session titled ‘Physiological Teachings of the Academician I.P. Pavlov on Psychiatry and Neuropathology’ was held in Moscow in October 1951. Sukhareva and several other prominent Russian psychiatrists, including Sukhareva’s supervisor Professor M.O. Gurevich and her colleague Professor A.S. Shmar’ian, were severely castigated for holding anti-Marxist views and espousing Western psychiatric theories [75]. Their work was labelled ‘anti-Marxist’, ‘anti-Pavlovian’, ‘reactionary’ and ‘idealistic’ and they were accused of perniciously impacting Soviet psychiatry. They were compelled to publicly confess that they had erred and were made to solemnly commit to only profess Pavlovian teaching in the future [93]. Shmar’ian’s school of brain pathology and neuropsychiatry was closed and as a result, Russia went without virtually any neuropsychiatric research for several decades [75].

Following this thorough excoriation, Sukhareva had cause to be anxious, although it appears that ultimately her career was not substantially damaged. One unverified account relates that in January 1953, after Stalin’s anti-Semitic ‘doctors’ plot’ campaign was in motion, Sukhareva left a large amount of banknotes at her friends, asking them to conceal it, so that her parents might be provided for, should she be arrested by the authorities [95]. It is also the case that Sukhareva had to self-censor her work and omitted non-Russian authors in the bibliography of her first volume of Clinical Lectures on Developmental Child Psychiatry. Leo Kanner related that in 1955 Sukhareva sent him a copy of this work. “This was during the Stalin regime”, Kanner wrote. “I was, of course, familiar with Dr. SSUCHAREWA’s work and had quoted her repeatedly, especially in connection with her valuable studies of childhood schizophrenia. I knew that she was well acquainted with the international literature. Yet in the 447 pages of text, there was not the slightest hint that anything had been done in the field by anybody not a native of the U.S.S.R….” he declared. “A second, revised edition appeared in 1959”, Kanner continued; “This was after Stalin had left the scene of action and the censors had begun to relent somewhat. This time the bibliography had 230 references; of these, 182 were Russian and 48 originated elsewhere (24 German, 12 French, 4 Swiss, and 2 each American, British, Italian and Austrian). Undoubtedly, Dr. SSUCHAREWA had been aware of those before but, in a somewhat relaxed political milieu, was able to acknowledge this publicly” [11, 18, 96]. Simmonds [11] observed that Kanner was partially mistaken, as Sukhareva had, in fact, referred to several non-Russian authors in the main body of her work but had not included them in the bibliography, which under the circumstances appears to have been a principled but somewhat hazardous strategy.^4^

## Modern recognition

It may be questioned why Sukhareva is only now granularly receiving the wide acclaim that should have followed her initial 1926 publication. Manouilenko and Bejerot [6] posit that Sukhareva’s gender, her Jewish identity, Russian nationality and Russian and German-language publications were not an efficacious recipe for gaining worldwide attention in the 1920s. Today, it is decried that “despite all” of Sukhareva’s pioneering contributions to understanding autism, her work “remains even today a kind of curiosity and it is only sporadically cited in literature” [30].

There have been calls from many quarters for greater recognition of Sukhareva’s pioneering role in first providing a clinical account of autistic children in 1926, almost two decades before Kanner and Asperger. Manouilenko and Bejerot [6] both of the Karolinska Institutet in Stockholm, argued that despite a Russian-language commemorative article appearing on the 120th anniversary of Sukhareva’s birth [74], much wider acknowledgement of her work is needed. In the Journal of Pediatric Neurosciences in 2017, Dr. Annio Posar and Paola Visconti of the University of Bologna opined “More than 20 years have passed since the release of the English translation of the original paper by Grunya Efimovna Sukhareva (Kiev, 1891–Moscow, 1981) entitled “Die schizoiden Psychopathien im Kindesalter,” but the international literature on autism has not yet given the right acknowledgment to this child psychiatrist who remains still unknown to many authors” [30].

Posar and Visconti’s letter to the editor made clear that recognition of Sukhareva as being the first to describe autism was necessary. In their composition, titled ‘Tribute to Grunya Efimovna Sukhareva, the woman who first described infantile autism’, it was observed that the ‘official history of autism’ ascribes the first descriptions of individuals who would be diagnosed today with autism spectrum disorder (ASD) to Kanner and Asperger. “However” they wrote, “already in 1926, Grunya Efimovna Sukhareva…who was then active in the Union of Soviet Socialist Republics, had described six boys presenting with a clinical picture that, as for the clinical features and evolution, is fully compatible, according to the modern criteria, with ASD and that today we would call “high functioning.”” Posar and Visconti affirmed that advances in modern understanding of autism present a more convoluted picture than that presented by Sukhareva.^5^ “But”, they asserted, “denying the originality and the accuracy of her report, more than 90 years after its release, would be a historic mistake, which we hope will be not perpetual” [30].

Certainly, in Russia, academics have written that Sukhareva “is rightfully considered the founder of child psychiatry in our country” [14]. Today, Sukhareva’s works remain key teaching material for students of child psychiatry in Russia [14, 74]. As already indicated, knowledge of her work is slowly spreading beyond the Russian Federation. One notable example is Simmonds’ translation of the 1927 sequel to Sukhareva’s 1926 paper, which provides five clinical case studies of autistic girls [12]. Beyond literature already adumbrated, international journal articles have recognised Sukhareva as being the first to provide detailed descriptions of children that match today’s conception of ASD [65, 97–101]. Since 2015, by order of the Moscow Department of Health, the Moscow Scientific and Practical Centre for Mental Health of Children and Adolescents, where Sukhareva worked for 40 years, was renamed the G. E. Sukhareva Scientific and Practical Centre for Mental Health for Children and Adolescents. A large commemorative plaque with a bust of Sukhareva’s profile adorns the building. The institution, which is the oldest of its kind in Russia, refers to Sukhareva as a “child psychiatrist of world importance” [35]. Alexander Goryunov, head researcher at the department, defined Sukhareva as the “most well-known name in child psychiatry” in Russia [9].

## Conclusion

A paper titled ‘G.E. Sukhareva: a course of life and scientific/pedagogical heritage (to the 125th anniversary of birth)’ appeared in the Russian-language Journal of Neurology and Psychiatry [74]. It was published in 2017, the year that the Department of Child Psychiatry and Psychotherapy of the Russian Medical Academy of Postgraduate Education marked 80 years since its foundation. That year also marked 125 years since the birth of the founder of the department, G.E. Sukhareva. Several of Sukhareva’s publications have also been translated over several decades into Chinese [102], English [5, 12], Spanish [103], German [5, 16, 17, 58, 63, 104–107], Polish [108–110] and French [111]. However, it is clear that more work needs to be executed. Translations of Sukhareva’s other publications is one avenue for future endeavours. Greater mindfulness of her wider psychiatric contributions, beyond her critical work on autism, is also needed. Overall, it remains essential for Sukhareva’s position as the first to publish clinical descriptions of autistic children to be more widely recognised. The focus on Kanner and Asperger alone in historical accounts of the genesis of describing, categorising, and naming autism has now been challenged with revelations indicating the pivotal role of Georg Frankl and Anni Weiss in this process. However, it is now equally pressing for Sukhareva’s pioneering contribution to modern understanding of autism to be more broadly acknowledged, for Sukhareva’s pioneering descriptions of autistic traits preceded those of Kanner, Frankl, Weiss, and Asperger by many years. In light of the unacceptable levels of stigma suffered by members of the autistic community, it is perhaps most fitting that the academic to first publish descriptions of autistic characteristics was a beloved female psychiatrist, a researcher who emphasised the manifold talents of her case studies at every opportunity.

## Data Availability

Not applicable.

## Footnotes

1 Pedology was a term current in Russia which referred to a discipline amalgamation of pedagogy, psychology and medicine.

2 There are further examples of Asperger’s anti-Semitism. Following the *Anschluss*, Jewish children in non-Jewish foster families were placed in Jewish orphanages and then transported to death camps. In March 1938, Asperger recommended separating a 13-year-old Jewish boy named Alfred from his non-Jewish foster mother -despite his affection for her- and placing him with Jewish foster parents; a highly questionable judgment. For further examples, see Czech [49], Sheffer [60] and Sher [61].

3 In 1917, Pavlov asked Vladimir Lenin for permission to transfer his laboratory overseas, a request that was refused. In 1923, Pavlov publicly denounced Communism and international Marxism. On Communism, he stated “For the kind of social experiment that you are making, I would not sacrifice a frog’s hind legs!” Three years later, in 1927, he wrote to Joseph Stalin “On account of what you are doing to the Russian intelligentsia—demoralizing, annihilating, depraving them—I am ashamed to be called a Russian!” When Nikolay Bukharin, Russia’s commissar of education, wished to visit Pavlov’s laboratory, Pavlov refused him entry, despite the disbursement of funds to Pavlov’s institution from Bukharin’s office [92, 94].

4 The second book Kanner received was not a second edition of the first book but Volume 2, an entirely new book. In fact, the first book Kanner had received in 1955 was a second edition of the first volume [11].

5 In particular, they state intellectual disability is often associated with ASD. It is unclear what is meant by this, for Sukhareva unambiguously referred to intellectual disability in her case studies.
